# A Compliant SMA-Actuated Capsule Robot with Integrated Locomotion and Steering for Wireless Capsule Endoscopy

**DOI:** 10.3390/mi17040471

**Published:** 2026-04-14

**Authors:** Ahmad M. Alshorman, Bashar Sh. Al-zu’bi, Omar A. Ababneh, Abdel Rahman Al Manasra, Khaled Alshurman, Tarik Alhmoud

**Affiliations:** 1 Department of Mechanical Engineering, Faculty of Engineering, Jordan University of Science and Technology, Irbid 22110, Jordan; bsalzubi200@eng.just.edu.jo (B.S.A.-z.); oaababneh19@eng.just.edu.jo (O.A.A.); 2 Department of General Surgery and Urology, Faculty of Medicine, Jordan University of Science and Technology, Irbid 22110, Jordan; aaalmanasra@just.edu.jo; 3 Electrical and Electronics Engineering Department, Hubei Polytechnic University in Huangshi, Huangshi 435000, China; 4 Gastroentetology and Hepatology Division, King Hussien Cancer Center, Amman 11118, Jordan

**Keywords:** wireless capsule endoscopy, capsule robotics, shape memory alloy actuation, compliant mechanisms, biomedical robotics

## Abstract

Wireless Capsule Endoscopy (WCE) is a minimally invasive technology for imaging the gastrointestinal (GI) tract, particularly the small intestine, where conventional endoscopy faces accessibility limitations. Traditional capsule endoscopes rely on passive motion driven by natural peristalsis, which limits controllability and may increase the risk of capsule retention. To address these challenges, this study presents the design and experimental validation of a compliant active capsule endoscope actuated by four Shape Memory Alloy (SMA) spring actuators. A key feature of the proposed system is a steering mechanism that reuses the same SMA actuators responsible for locomotion, enabling control of the camera orientation without increasing system complexity, size, or weight. The capsule architecture consists of rigid polylactic acid (PLA) links connected through thermoplastic polyurethane (TPU) flexure hinges, fabricated using dual-material 3D printing. Nonlinear finite element analysis (FEA) was employed to optimize the flexure hinge geometry for maximum displacement while maintaining safe stress levels. To validate the concept, a 3.5× scaled prototype was fabricated and integrated with SMA actuators and an Arduino-based control system. The experimental results demonstrate effective locomotion and steering capabilities, achieving a maximum stroke of approximately 5.4 mm and a steering angle of 24° for the 3.5× scaled prototype, corresponding to an estimated stroke of approximately 1.98 mm (Based on the FEA) at the intended clinical scale. Thermal characterization of the SMA actuators was also conducted to identify suitable operating current ranges for future biomedical deployment. The results demonstrate the feasibility of integrating locomotion and steering within a compact compliant capsule architecture, representing a step toward next-generation capsule endoscopy systems with improved navigation and diagnostic capability.

## 1. Introduction

Diseases of the gastrointestinal (GI) tract represent a major global health burden, with conditions such as colorectal cancer and Crohn’s disease contributing significantly to morbidity and mortality worldwide. Accurate and comprehensive visualization of the GI tract is therefore essential for early diagnosis and effective treatment. Traditional endoscopy using flexible tubes, while effective in many cases, often fails to reach critical regions of the esophagus, stomach, and small intestine. Moreover, the discomfort and invasiveness associated with conventional procedures deter many patients from undergoing routine screening, further undermining early detection efforts [1,2,3].

A significant breakthrough occurred in 2000 with the introduction of Wireless Capsule Endoscopy (WCE) [4], which received FDA approval in 2001 and was commercialized as the PillCam SB [5]. This swallowable robotic device provided a reliable and minimally invasive alternative to traditional endoscopy, dramatically expanding the diagnostic reach of clinicians while improving patient acceptance [6]. Since its introduction, capsule endoscopy has revolutionized small bowel imaging and continues to evolve toward active, intelligent devices capable of precise navigation, real-time localization, and targeted diagnosis.

Most capsule endoscopes used today are passive, meaning they rely on the natural movements of the gastrointestinal tract. Commercial examples such as PillCam SB3^®^ measure 26 mm in length, 11 mm in diameter, weigh approximately 4 g, provide a 12 h battery life, and capture images at 2–20 frames per second [7]. The main components of capsule endoscopy in commercial capsules generally include a vision module (camera, lens, light source), most often using CMOS sensors [8]; a wireless data transmission system (since onboard storage is impractical) [9]; and a power source.

Despite their clinical utility, passive capsules have notable limitations, such as unpredictable movement and the risk of retention due to reliance on GI peristaltic movements [10,11]. Active capsule endoscopy seeks to overcome these challenges by enabling controlled propulsion, thereby improving diagnostic reliability and safety [12]. External actuation systems address internal power and space constraints by using external magnetic fields to manipulate capsules containing internal magnets [13]. Electromagnetic actuation (EMA) systems, for instance, employ external coils to guide capsule motion and orientation [14,15], while permanent magnets can be maneuvered via robotic arms or handheld devices [16].

Internally actuated capsules incorporate locomotion mechanisms, actuators, and attachment strategies. Locomotion methods include friction-based designs (inchworm mechanisms [17] and legged approaches [18,19]), hydrodynamic swimming for fluid environments [20], and vibration-based methods to reduce friction and enhance control [21]. Actuator selection is critical for achieving sufficient force with low power consumption; common types include Shape Memory Alloys (SMAs) [22,23,24], DC motors [25], piezoelectric elements [26], and Ionic Polymer–Metal Composites (IPMCs) [27,28]. Attachment mechanisms are often bio-inspired, such as elastomeric micro-pillar adhesives that provide temporary, non-damaging adhesion to the GI wall [12,29].

Hybrid systems combine external and internal locomotion, using external magnets in open areas and internal mechanisms for collapsed or distended sections [30]. Another approach is electrical stimulus, which involves the use of electrical signals to make muscles contract, either moving or stopping the capsule. This method promises better speed, direction control, and lower power use [31]. Recent developments in robotic capsule endoscopy have emphasized the importance of improving the navigation, therapeutic capabilities, and intelligent control of the capsule robot; for example, magnetically actuated capsule robots have been proposed for the purpose of navigating and carrying out interventions in the gastrointestinal tract. Chen et al. proposed a magnetically actuated robotic capsule for navigation and drug delivery, emphasizing the possibility of developing capsule robots with combined diagnostic and therapeutic capabilities [32]. Similarly, Zheng et al. proposed a magnetically controlled capsule robot for intestinal biopsy, emphasizing the possibility of developing capsule robots with combined capabilities for navigation and tissue sampling [33].

Recent research has emphasized the importance of developing multifunctional capsule robots with advanced diagnostic and therapeutic capabilities. For example, Cao et al. conducted a comprehensive review of the recent developments in robotic capsule endoscopy, emphasizing the importance of intelligent navigation, micro-actuation mechanisms, and therapeutic modules in robotic capsule endoscopy [34]. In addition, the possibility of developing dual-functional capsule robots with combined drug delivery and biopsy capabilities has been proposed for the purpose of expanding the application of capsule endoscopy beyond imaging [35].

Recent advances in active capsule endoscopy and compliant medical robotics have focused on improving locomotion efficiency, miniaturization, and integrated actuation [36]. SMA-based capsule robots and thermally driven actuation mechanisms have been investigated due to their compact size and high force-to-volume ratio. In addition, compliant mechanisms have been widely adopted in medical microrobots to reduce mechanical complexity and improve reliability. Recent studies have also explored hybrid locomotion strategies, intelligent control, and AI-driven navigation for capsule robots operating in complex gastrointestinal environments [37,38,39,40]. These developments highlight the need for compact designs that integrate locomotion and steering using minimal actuators while maintaining structural simplicity, adaptability, and scalability [41].

While existing research on active capsules aims to address the general limitations of passive capsules, many of these solutions still face challenges; for instance, capsules that utilize external actuation systems can provide controlled movement and navigation. However, this approach often requires patients to stay in a hospital environment and incurs high costs, as it relies on large external magnetic sources or robotic arms for capsule control. Such dependencies limit the practicality of these methods for widespread clinical use.

In contrast, many internally actuated capsule designs found in the literature tend to be overly complex, incorporating many components that complicate both implementation and manufacturing. Additionally, most of these designs focus primarily on solving the retention issues of passive capsules, without providing mechanisms for directional control or tip orientation. This limitation considerably hampers their potential for advanced applications, such as accessing hard-to-reach areas, enabling targeted drug delivery, or performing surgical excisions.

This study proposes a novel and efficient design of an active capsule endoscope that incorporates a compliant mechanism for both locomotion and steering using Shape Memory Alloy (SMA) actuators. The proposed design adopts a compact architecture consisting of rigid links and flexible elements, enabling efficient deformation and motion within a constrained space. The capsule is actuated using internal SMA springs that generate the required motion through controlled contraction. A key feature of the proposed design is the integration of locomotion and steering functionalities using the same set of SMA actuators. Unlike conventional capsule robots that require separate mechanisms for steering and propulsion, the proposed approach eliminates the need for dedicated steering components. This integrated actuation strategy reduces the number of actuators, simplifies the mechanical architecture, minimizes the capsule’s overall size and weight, and enhances system reliability. Furthermore, the proposed design provides a flexible platform for future extensions, including targeted drug-delivery applications. The design builds upon and improves the mechanism introduced in [41], offering enhanced functionality through the unified actuation approach. To the best of the authors’ knowledge, this work represents one of the few active capsule endoscope designs that achieve both locomotion and steering using a single actuation mechanism.

Table 1 compares the proposed capsule robot with representative active capsule endoscopy systems reported in the literature. Many existing designs rely on external magnetic actuation, which requires large electromagnetic systems and clinical infrastructure for capsule navigation. Other internally actuated capsules employ separate mechanisms for locomotion and steering, increasing mechanical complexity and internal volume requirements. In contrast, the proposed capsule implements both locomotion and steering using the same set of SMA spring actuators integrated within a compliant mechanism architecture. This design reduces mechanical complexity while achieving a locomotion stroke of approximately 5.4 mm and a steering angle of 24°, demonstrating the feasibility of a compact internally actuated capsule robot.

Table 1 compares representative capsule endoscopy systems in terms of actuation method, locomotion capability, and system complexity. Passive capsule systems rely on gastrointestinal peristalsis and provide no locomotion or steering capability. Internal actuation approaches, including vibratory motors and DC motor-driven mechanisms, enable active locomotion but typically increase mechanical complexity due to gears, linkages, and multi-part assemblies. Hybrid and externally actuated magnetic systems provide improved steering and continuous navigation; however, they require bulky external hardware such as robotic magnetic platforms or electromagnetic coil systems, limiting portability and clinical scalability. In contrast, the proposed capsule integrates locomotion and steering using a compliant mechanism driven by shared SMA actuators, eliminating transmission components and external actuation hardware. This significantly reduces mechanical complexity while maintaining active locomotion and directional control within a compact capsule size. The simplified architecture and fabrication approach highlight the potential of compliant SMA-based designs for scalable and clinically deployable active capsule endoscopy.

The major contributions of this work can be highlighted as follows:

- Design of a compliant active capsule endoscope architecture that incorporates both locomotion and steering functionalities using the same set of shape memory alloy (SMA) spring actuators, thereby eliminating the need for steering mechanisms.
- Optimization of the flexure hinge design using nonlinear finite element analysis (FEA) to maximize the achievable locomotion stroke while ensuring the structural integrity and safe stress levels of the compliant mechanism.
- Achievement of a locomotion stroke of 5.4 mm and a steering capability of 24°, thereby offering improved maneuverability compared to several internally actuated capsule endoscope mechanisms reported in the literature.
- Design and integration of a haptic control interface that allows the operator to orient the capsule endoscope tip, thereby facilitating the intuitive control of the viewing direction.
- Experimental validation of the designed compliant active capsule endoscope architecture, which involves locomotion, steering, and thermal characterization of the SMA actuators for determining the safe operating current ranges.

## 2. Proposed Capsule Design and Working Principle with Flexure Hinge Mechanism

**Figure 1:**
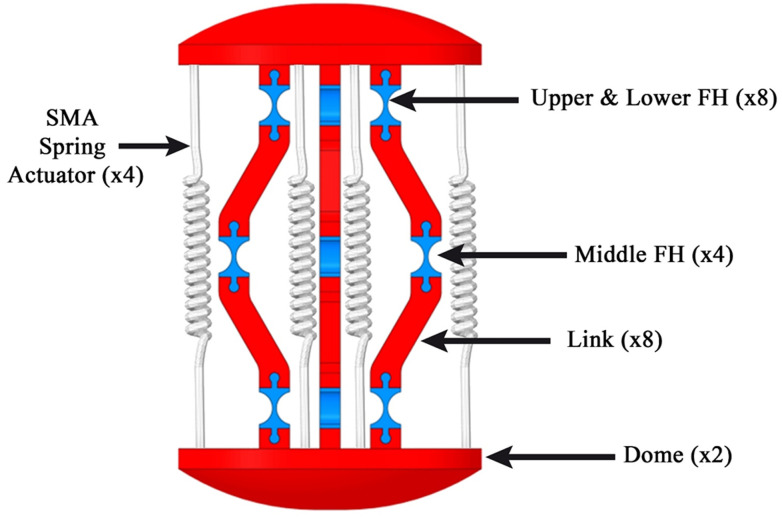
The main structure of the capsule.

**Figure 2:**
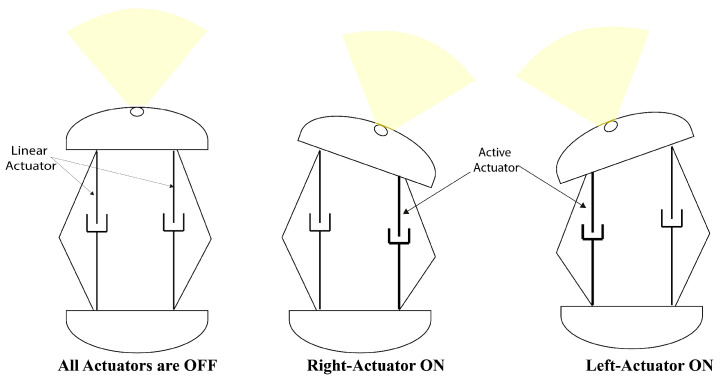
Camera’s steering mechanism.

**Figure 3:**
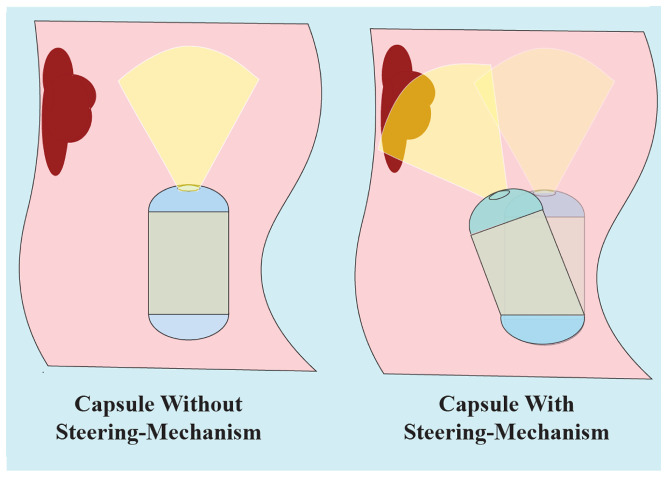
The Steering mechanism is used to increase the camera view angle.

**Figure 4:**
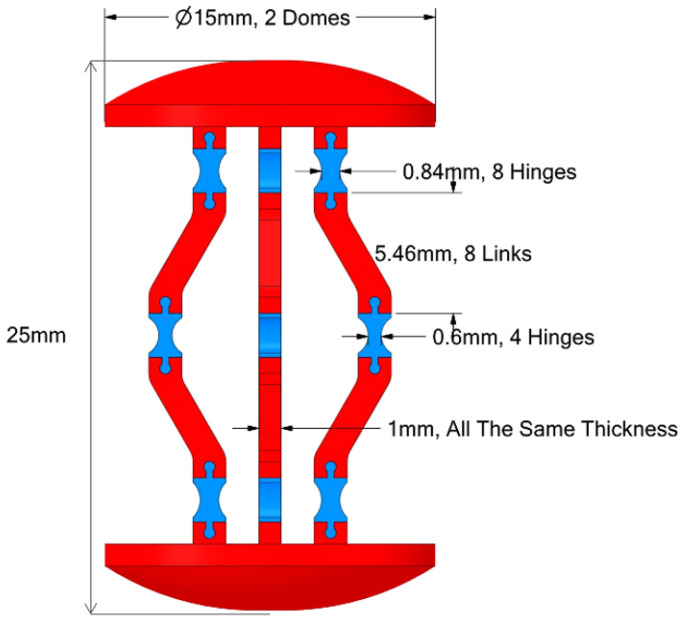
The capsule’s optimized dimensions.

The proposed capsule endoscope signifies a substantial advancement in minimally invasive medical technology, distinguished by its innovative parallel manipulator architecture. As illustrated in Figure 1, the system is composed of eight rigid links connected by twelve flexible joints and features two domes. The design incorporates four shape memory alloy (SMA) springs positioned between the front and rear domes, which facilitate both locomotion and precise manipulation of the camera, as demonstrated in Figure 2. This dual functionality enhances the viewing angles during diagnostic procedures, particularly for the inspection of pathological lesions and the execution of targeted interventions (Figure 3). With compact dimensions of 25 mm in length and 15 mm in diameter (Figure 4), the capsule efficiently adheres to biological constraints while housing the essential mechanical components.

The motion system is based on a sophisticated semi-compliant mechanism that combines rigid PLA structural elements with precisely engineered TPU flexure hinges. This hybrid design capitalizes on the benefits of compliant mechanisms while preserving necessary structural integrity. A comparative analysis of several notch hinge configurations has unveiled distinct performance characteristics tailored to fulfill diverse functional requirements within the system. The parallel manipulator architecture provides two controlled degrees of freedom: roll about the X-axis through coordinated activation of Actuators 1 and 3, and pitch about the Y-axis via Actuators 2 and 4.

**Figure 5:**
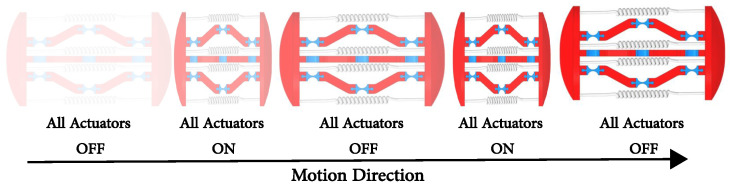
Proposed capsule actuation principle.

The actuation principle employs a three-stage process (Figure 5), commencing from a rest state with deactivated SMA actuators. The process advances to active manipulation through selective electrical activation of specific springs, culminating in recovery facilitated by the shape memory effect once the electrical current is ceased.

This design not only enhances the operational capabilities of the capsule endoscope, but also aligns with the overarching objective of improving diagnostic accuracy and patient comfort during minimally invasive procedures.

## 3. Finite Element Analysis and Results

Material selection is crucial in the capsule’s design, with an emphasis on mechanical performance and biocompatibility. The thermoplastic polyurethane (TPU) 92A was selected for its flexibility, exhibiting a Young’s modulus of 15.3 MPa for the construction of the flexure hinges (FHs). In contrast, polylactic acid (PLA) was chosen for its structural rigidity, having a Young’s modulus of 3450 MPa, to fabricate the dome and link components, as outlined in Table 2.

**Figure 6:**
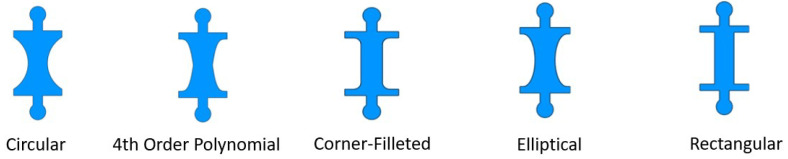
Different notch shapes of the flexure hinges.

To optimize the capsule’s performance, a nonlinear finite element analysis (FEA) was performed using ANSYS 2021 R1 Workbench, which accounted for the material nonlinearity and the large deformations of the capsule. This analysis involved the systematic evaluation of several notch shapes of hinge geometries, as depicted in Figure 6. Key factors considered included maximum Von Mises stress, edge deformation, and rotational axis deformation.

**Figure 7:**
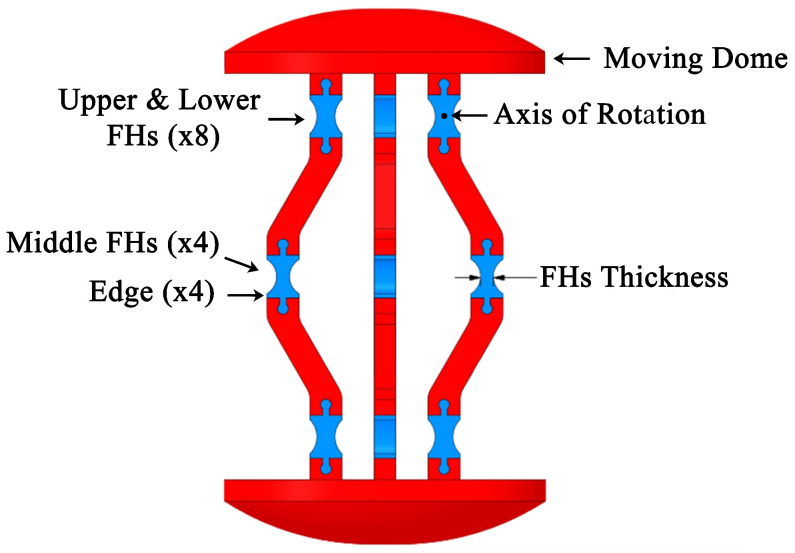
Main parts of the optimization.

Thickness optimization studies, summarized in Table 3 and illustrated in Figure 7, resulted in design parameters that effectively balanced mechanical stress limitations with the functional displacement requirements.

**Figure 8:**
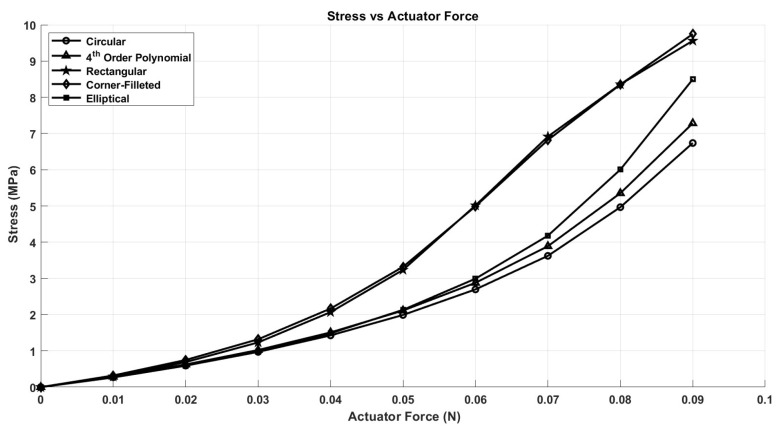
The stress of different notch shapes.

**Figure 9:**
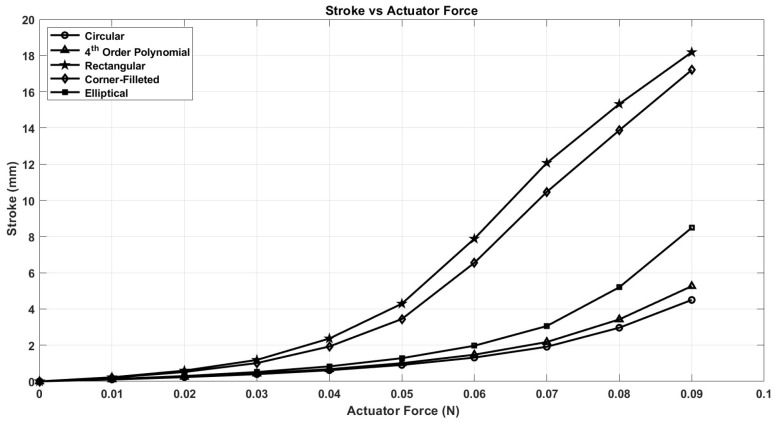
Stroke of different notch shapes.

**Figure 10:**
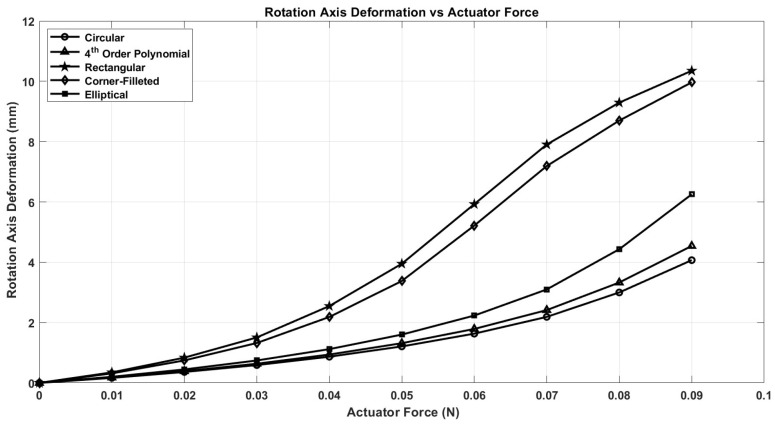
Rotation axis deformation for different notch shapes.

After conducting nonlinear finite element analysis (FEA) simulations on several notch geometries using ANSYS Workbench, we present the results for five distinct notch shapes: circular, elliptical, rectangular, corner-filleted, and 4th-order polynomial. These results are illustrated in Figure 8, Figure 9 and Figure 10.

In Figure 8, it is evident that under equivalent actuator forces, the stroke value of the capsule is maximized with the rectangular notch shape and minimized with the circular notch shape. At first glance, this suggests that the rectangular shape may be the superior option; however, several other factors must be considered to determine the optimal notch shape. One notable factor is the stress distribution associated with each notch design. The circular notch shape shows a lower stress profile for a given input force when compared to the other geometries evaluated. This advantage arises from its smooth, continuous curvature, which minimizes stress concentration points that are typically present in shapes with sharp corners. Conversely, rectangular notches are prone to stress concentration due to their geometric discontinuities; the sharp corners of these notches catalyze localized areas of high stress, thus increasing the likelihood of structural failure.

Another critical consideration is the robustness of the capsule design. The capsule featuring the circular notch shape exhibits superior robustness compared to its rectangular counterpart, enabling the force-holding (FH) structures to store greater potential energy and facilitate smoother motion of the capsule. This increase in robustness is reflected in the requirement for a greater force to achieve the same stroke in the circular notch shape, as illustrated in Figure 9.

The deformation experienced at the axis of rotation of the middle FH structures, termed axis shift, is an additional factor influencing the choice of notch shape, as it directly impacts the accuracy of the capsule’s dynamic model. It is important to note that accuracy improves with a decrease in axis shift. As demonstrated in Figure 10, the circular notch shape exhibits the lowest axis shift, in stark contrast to the higher values observed in the rectangular notch shape. Given these considerations, we have concluded that the circular notch shape is optimal for our design. Subsequently, we applied the Response Surface Optimization toolbox in ANSYS to refine the thickness of the capsule components. The optimized thickness obtained is 0.88 mm for both the upper and lower FHs, and 0.52 mm for the middle FHs, as detailed in Table 3.

**Figure 11:**
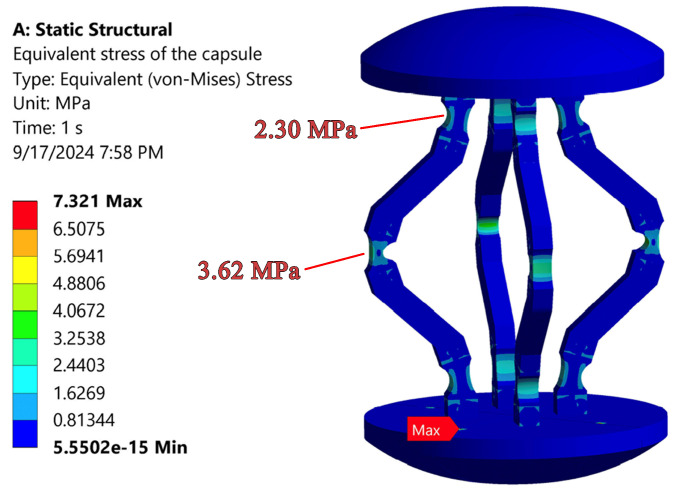
The Von-Mises stress of the capsule with Optimized Geometry.

The optimal design configuration constrains the maximum equivalent force applied by each shape memory alloy (SMA) actuator in the finite element model to 0.092 N, thereby ensuring that the edges remain within the confines of the capsule structure. The von Mises stress distribution for this optimal capsule design, evaluated at the maximum force, is depicted in Figure 11. As anticipated, the maximum von Mises stress is concentrated at the middle FHs, yielding a safety factor of 1.56 and a maximum deformation of the moving dome of 1.98 mm. Furthermore, the finite element analysis results indicate that the stress experienced by the upper and lower FHs is 2.3 MPa, while the middle FHs experiences a stress of 3.62 MPa, as illustrated in Figure 11.

The equivalent actuator force used in the FEA was determined by applying a gradually increasing input force to the compliant mechanism until the design constraints were reached. These constraints included the allowable material stress based on the yield stress with an applied safety factor, as well as the maximum permissible edge deformation. The force value at which these limits were satisfied was identified as the allowable actuator force. This resulted in an equivalent force of 0.092 N for the original capsule-scale model.

The relationship between this equivalent force and the actual SMA output was established using the experimental characterization of the SMA springs. The maximum current that can be applied to the SMA without exceeding the allowable force corresponds to approximately 3 A for the 3.5× scaled prototype. It should be noted that the 0.092 N value corresponds to the FEA conducted at the original capsule size, whereas the experimentally measured current–force relationship was obtained using the scaled prototype. Accordingly, the selected operating current ensures that the SMA-generated force remains within the allowable structural limits defined by the FEA.

## 4. Kinematic of the Proposed Capsule Endoscopy

**Figure 12:**
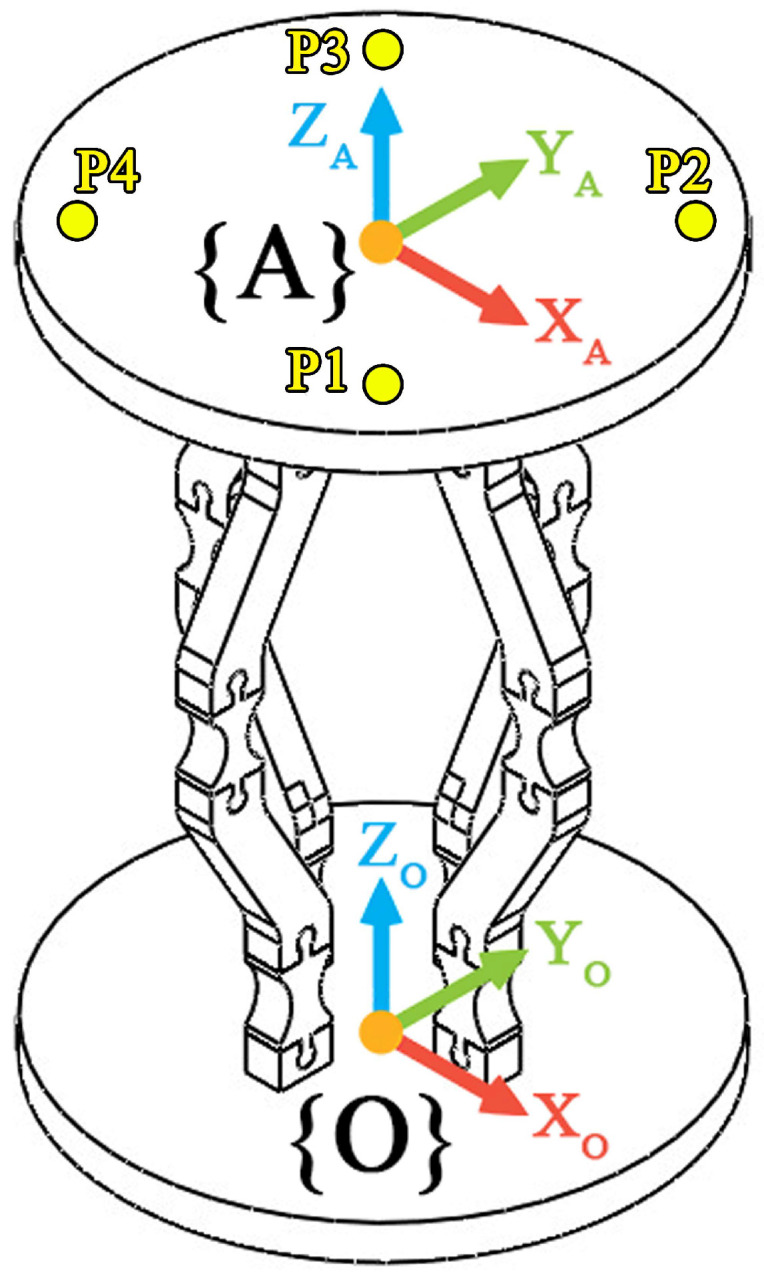
Attached frames and points at a fixed base and a moving platform.

The motion system of the capsule is designed using an advanced semi-compliant mechanism that integrates rigid PLA structural components with meticulously crafted TPU flexure hinges. This hybrid configuration leverages the benefits of compliant mechanisms while ensuring the required structural stability. A comparative evaluation of different notch hinge designs indicated that circular hinge geometries provide enhanced rotational accuracy, achieving a resolution of ±0.5°, whereas corner-filleted designs allow for greater angular displacements, reaching up to 12°. This diversity makes both hinge types suitable for several functional needs within the same system. The schematic representation of the parallel manipulator architecture is depicted in Figure 12.

**Figure 13:**
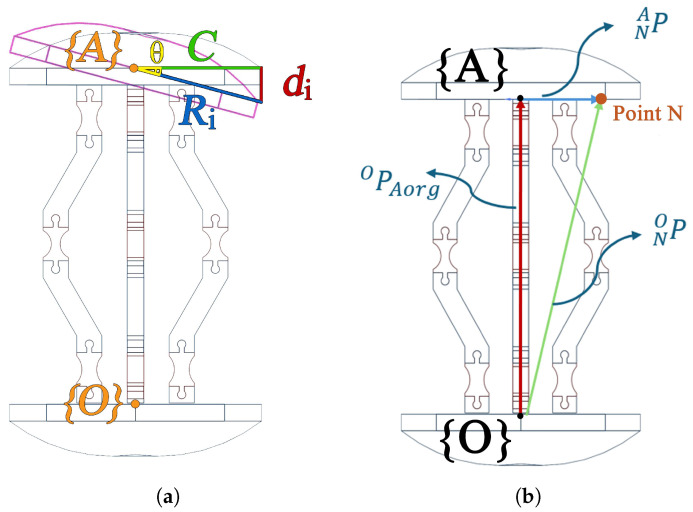
Side view of proposed capsule (**a**) Capsule side view. (**b**) Position vectors.

The kinematic analysis of the system is conducted using a robust mathematical framework that links the deformations of the SMA wires to the orientation of the platform. The forward kinematics solution, represented by Equation (1) and illustrated in Figure 13a, determines the platform’s pose based on the displacements of the actuators. This transformation is accomplished through an integration of position vectors (Equations (2)–(4)) and rotation matrices (Equations (5)–(7)), which together delineate the spatial relationship between the fixed base frame {O} and the moving platform frame {A}, as shown in Figure 13b. The inverse kinematics solution (Equation (12)) allows for precise control by calculating the necessary actuator deformations to achieve specified platform orientations. This comprehensive kinematic model addresses the intricate interactions among multiple actuators while ensuring accuracy, performance.

(1) $$\theta =tan^{-1}\left(d_{i}/C\right)$$

where $d_{i}$ represents the contraction of the *i*-th SMA actuator and *C* denotes the geometric distance between the actuator attachment point and the rotational axis of the capsule platform. The kinematic model establishes the relationship between the contraction of each SMA actuator and the resulting angular orientation of the capsule platform. The displacement of the *i*-th actuator, denoted by $d_{i}$, determines the rotation angle θ of the capsule platform as expressed in Equation (1). This relationship captures how the actuator deformation is translated into the angular motion of the front dome carrying the camera.

By combining the position vectors defined in Equations (2)–(4) with the rotation matrices presented in Equations (5)–(9), the spatial orientation of the moving platform can be determined relative to the fixed base frame. This formulation allows the transformation between actuator displacements and the orientation of the capsule tip.

Consequently, the kinematic model provides the mathematical framework required to map actuator contractions into the desired platform orientation. This relationship is essential for controlling the steering of the capsule, as it enables the computation of the actuator displacements required to achieve a specified viewing direction of the camera. In practical implementation, this model can be used within the control algorithm to compute the required actuator inputs corresponding to a desired steering angle of the capsule.

Here,

- N: The point number.
- PAorgO: Position vector from {A} to frame {O}, given by

(2) PAorgO=PAXOPAYOPAZO

- PNA: Position vector from point N to frame {A}, given by

(3) PNA=PNXAPNYAPNZA

- PNO: Position vector from point N to frame {O}, given by

(4) PNO=PNXOPNYOPNZO

- Rotate frame {A} about XO-axis by angle (γ):

(5) RXOA(γ)=1000cos(γ)−sin(γ)0sin(γ)cos(γ)

- Rotate frame {A} about YO-axis by angle (β):

(6) RYOA(β)=cos(β)0sin(β)010−sin(β)0cos(β)

- Rotate frame {A} about ZO-axis by angle (α):

(7) RZOA(α)=cos(α)−sin(α)0sin(α)cos(α)0001

(8) RXYZOA(γ,β,α)=RZ(α)RY(β)RX(γ)=cos(α)−sin(α)0sin(α)cos(α)0001cos(β)0sin(β)010−sin(β)0cos(β)1000cos(γ)−sin(γ)0sin(γ)cos(γ)

(9) RXYZOA(γ,β,α)=cosαcosβcosαsinβsinγ−sinαcosγsinβcosγsinαcosβsinαsinβsinγ+cosαcosγsinαsinβcosγ−cosαsinγ−sinβcosβsinγcosβcosγ

After finding the position vectors and rotation matrices, we can now find the transformation matrix, given by

(10) TOA=ROAPAorgO0001

Hence, to find the position vector of each of the four points, multiply the position vector of each point by its corresponding transformation matrix.

(11) PON1=TAOPOA1

(12) $$d_{i}=C*tan\left(\theta \right)$$

## 5. Experimental Work and Validation

**Figure 14:**
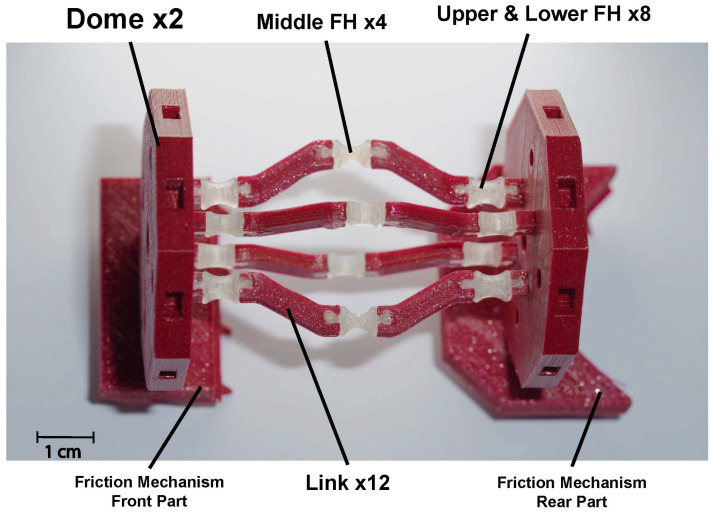
The fully assembled scaled capsule with its main components.

**Figure 15:**
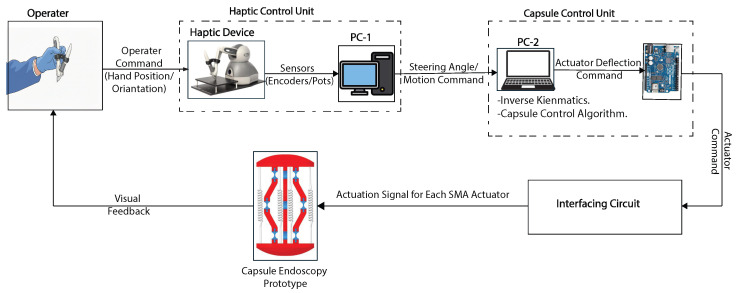
The block diagram of the capsule endoscopy system.

**Figure 16:**
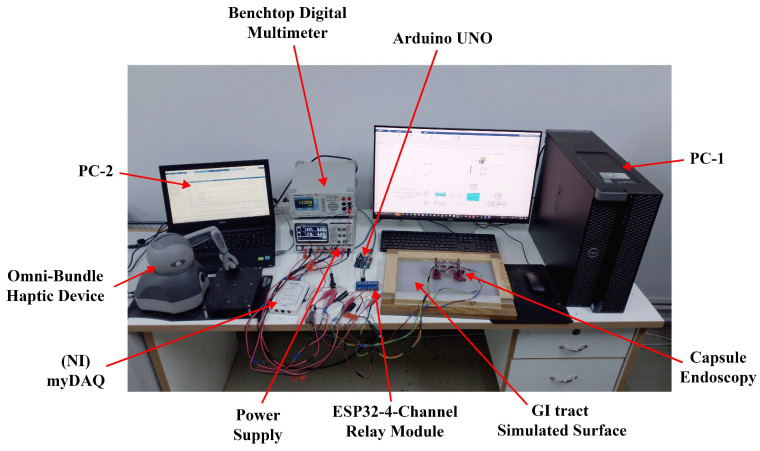
The experimental setup of the capsule system.

A prototype of the capsule was fabricated using advanced dual-material fused deposition modeling (FDM) techniques. This manufacturing process achieved an optimal balance between print quality and mechanical performance by using specific parameters, including a layer height of 0.2 mm, an infill density of 20% for the PLA components, and 100% infill for the critical TPU flexure hinges, as detailed in Table 4. The individual 3D-printed components were assembled into the final capsule prototype, as illustrated in Figure 14. The actuation system employs nickel–titanium–copper (NiTiCu) shape memory alloy springs, which exhibit consistent performance characteristics, achieving a contraction from 28 mm to 12 mm at an activation temperature of 70 °C. Figure 15 presents the block diagram of the capsule endoscopy system used in the experimental validation. The system consists of the compliant capsule mechanism actuated by four SMA springs, a control unit, a power supply module, and a measurement system. The control unit, implemented using an Arduino platform, generates actuation signals to drive the SMA springs in predefined sequences to achieve locomotion and steering. The power supply delivers controlled current levels required for SMA activation. The measurement system is used to record displacement and angular motion during the experiments. This block diagram illustrates the interaction between the electrical control system, actuation mechanism, and mechanical motion of the capsule. Figure 16 shows the full experimental system with its main components and the setup used for validation. The system includes the 3.5× scaled compliant capsule prototype, SMA spring actuators, a supporting frame, an Arduino-based control unit, an external power supply, and measurement tools. The capsule is mounted on a test platform that allows controlled actuation of the SMA springs to generate locomotion and steering motions. Displacement and steering angle were measured using calibrated visual references. This experimental setup was used to evaluate the kinematic performance, locomotion stroke, and steering capability of the proposed capsule design.

The present prototype uses an external power supply and Arduino-based control hardware for proof-of-concept validation. For a future wireless capsule implementation, power consumption can be reduced by sequential activation of the SMA actuators, short pulsed actuation, and duty-cycle control, such that only the actuators required for a specific locomotion or steering task are energized at a given time. In addition, future designs may incorporate miniature high-energy-density batteries and, where appropriate, wireless power transfer to support autonomous operation.

**Figure 17:**
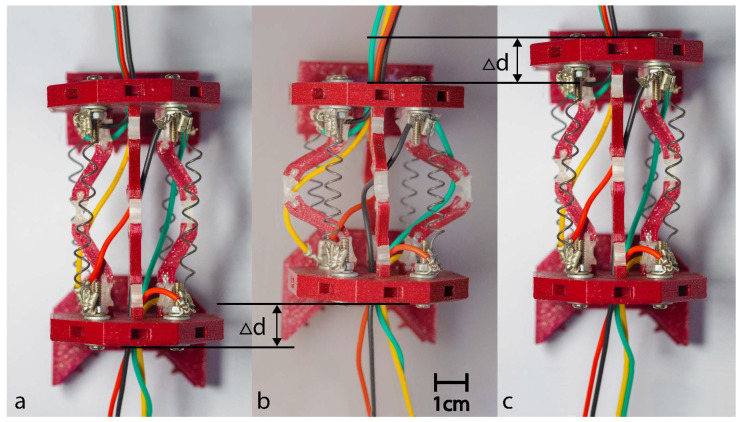
(**a**–**c**) Sequential frames illustrating the forward locomotion cycle of the proposed capsule prototype.

**Figure 18:**
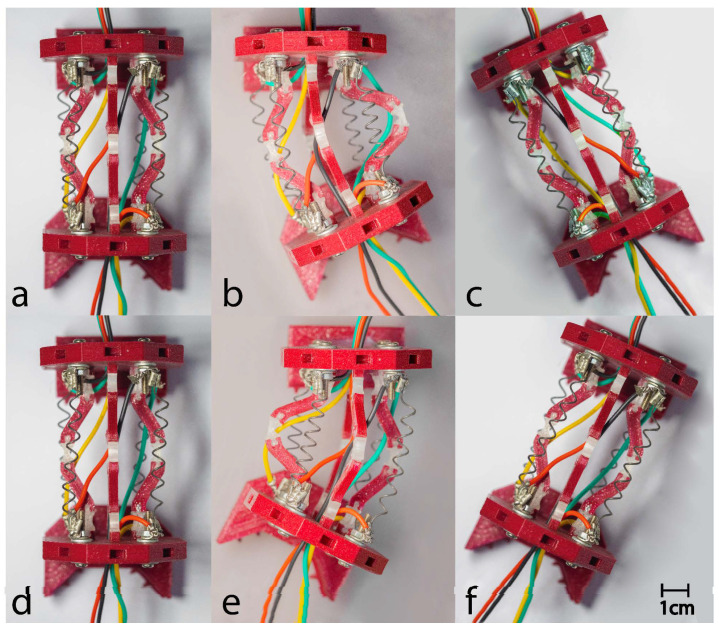
(**a**–**f**) Sequential frames illustrating the rotational motion cycle of the proposed capsule prototype.

**Figure 19:**
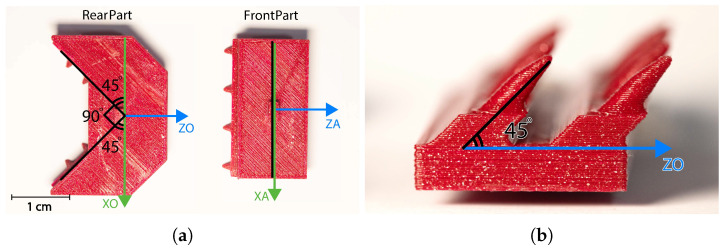
(**a**) Friction mechanism parts. (**b**) Pins angle.

The integrated capsule system comprised a mechanical platform paired with an Omni Bundle haptic interface for operator control. Experimental evaluations were conducted on a silicone-rubber surface, as illustrated in Figure 16. Forward motion was successfully achieved through the alternating activation of shape memory alloy (SMA) actuators, yielding consistent displacements of 5 mm (refer to Figure 17). Additionally, rotational motion was facilitated by selectively activating unilateral actuators, allowing for directional adjustments of 15° (see Figure 18). To enhance clinical applicability, the system demonstrated precise manipulation capabilities by coordinating the activation of SMAs to vary the dome’s viewing angles. The stability of anchoring during these movements was ensured by the angled pin friction mechanism (illustrated in Figure 19a,b). The angled pin friction mechanism shown in Figure 17 is used to provide directional anchoring during locomotion. The pins are oriented at approximately 45° relative to the motion direction, allowing them to generate asymmetric friction forces. When the capsule moves forward, the pins create higher resistance in the backward direction while allowing forward sliding. This directional friction enables one section of the capsule to anchor while the opposite section moves when SMA actuators are activated. The locomotion is achieved through sequential activation of the SMA springs, producing alternating contraction and extension of the compliant structure. During this process, one end of the capsule remains anchored by the angled pins while the other end advances, resulting in a walking-type motion. The rounded pin tips and distributed arrangement ensure stable anchoring while minimizing potential surface damage and reducing excessive resistance during steering and forward motion.

Comprehensive data acquisition was performed using an NI myDAQ (195509F-01L, Penang, Malysia) for voltage measurements across SMA springs and a FLIR thermal camera for monitoring temperature variations. Additionally, Tracker software (Ver 6.3.1) enabled frame-by-frame analysis of both displacement and velocity. The assembled experimental setup (shown in Figure 16) verified the reliable performance of the system across all modes of motion, while identifying potential opportunities for further precision enhancement.

The experimental prototype used in this study was manufactured at approximately 3.5× the scale of the intended final capsule design. This larger scale was chosen to make rapid prototyping easier, simplify assembly, and allow more accurate measurement of displacement, temperature, and actuator behavior during the experimental validation stage. Although the prototype is larger than a clinically deployable capsule, the core mechanical architecture, compliant mechanism behavior, and actuation principle remain representative of the proposed design.

It is important to note that scaling influences several physical parameters, such as actuator force requirements, thermal characteristics, and structural stiffness. In particular, miniaturizing the capsule will require smaller SMA actuators and careful thermal management to ensure safe operation in biological environments. Despite these differences, the kinematic relationships, compliant mechanism behavior, and actuation strategy demonstrated in the scaled prototype still provide a valid proof-of-concept for the proposed capsule architecture. Future work will focus on further miniaturization and the integration of micro-scale actuators suitable for biomedical applications.

**Figure 20:**
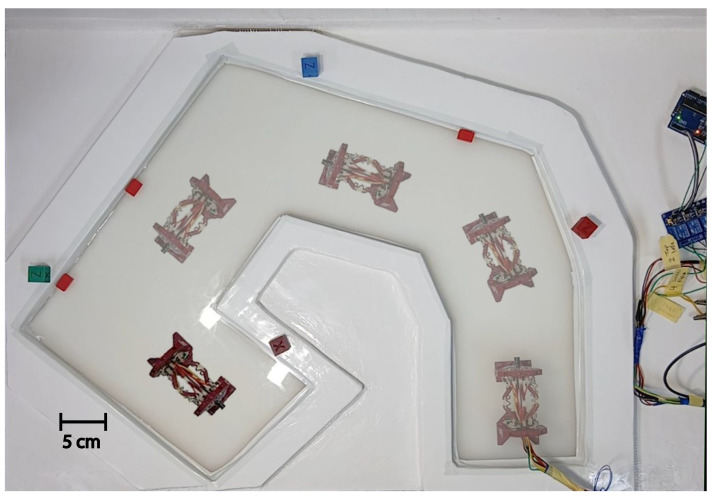
Capsule motion in a non-uniform path demonstrating steering and locomotion capability using SMA-actuated compliant mechanism.

Figure 20 illustrates the motion of the proposed capsule mechanism along a non-uniform path. AnThe experiment was conducted to evaluate the ability of the compliant mechanism to adapt to curved and irregular trajectories similar to those encountered in the gastrointestinal tract. By sequentially activating the SMA springs, the capsule generates asymmetric deformation of the compliant links, resulting in directional locomotion and steering. The images show that the capsule can successfully follow a curved path while maintaining stable contact with the surface. This behavior demonstrates the capability of the integrated locomotion and steering mechanism to navigate non-uniform environments without requiring additional steering actuators.

**Figure 21:**
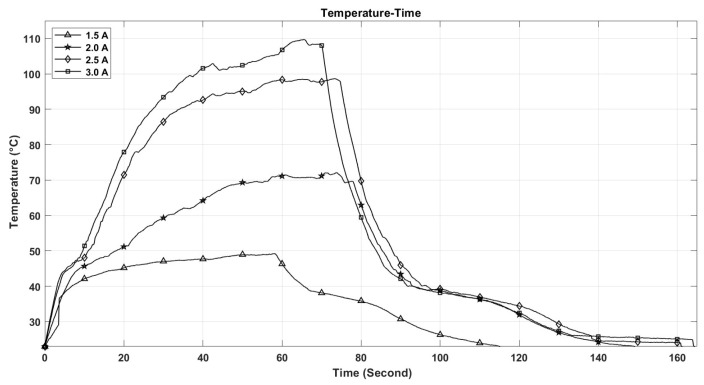
The shape memory alloy actuator temperature vs. time at different DC current inputs.

**Figure 22:**
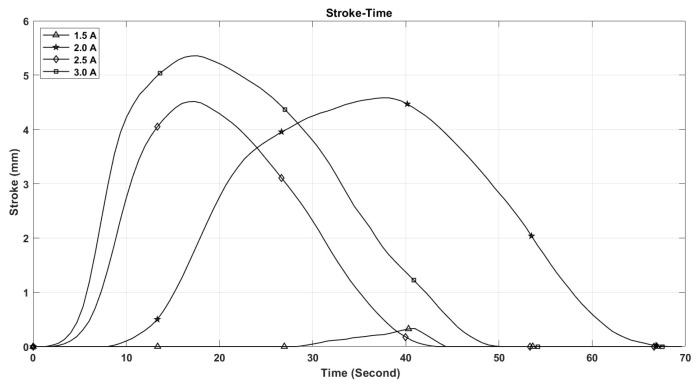
The capsule stroke vs. time at different current values.

**Figure 23:**
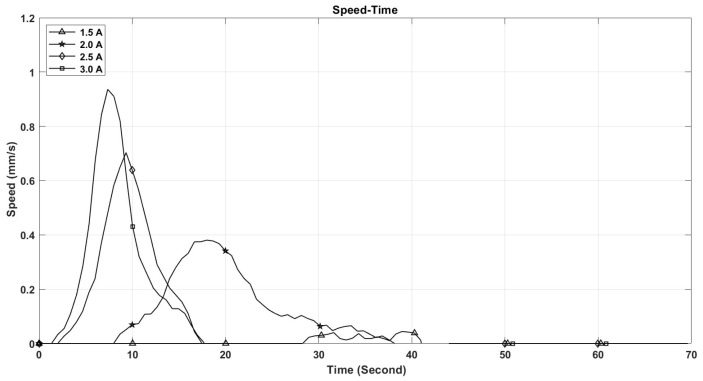
The capsule speed vs. time at different current values.

**Figure 24:**
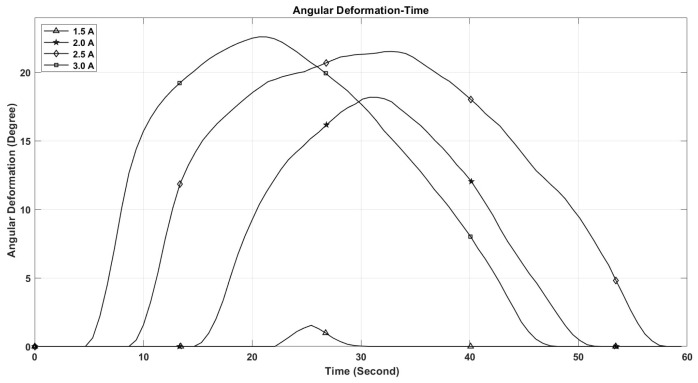
The angular deformation of the front dome with time at different current values.

The performance of shape memory alloy (SMA) actuators is significantly influenced by their operating temperature. This temperature is directly related to the electric current flowing through the actuator. As depicted in Figure 21, the maximum temperature of the SMA spring increases with the current intensity, reaching 110 °C at 3 A, while remaining below 50 °C at 1.5 A. Furthermore, the rate at which the temperature of the SMA spring escalates is greater with higher current levels, as illustrated in Figure 21. This phenomenon accounts for the observed enhancements in stroke, speed, and angular deformation of the actuator with increasing current, as shown in Figure 22, Figure 23 and Figure 24.

**Figure 25:**
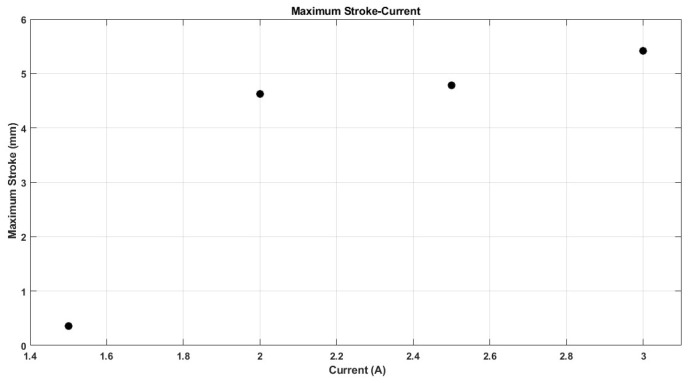
Maximum capsule stroke at each current value.

**Figure 26:**
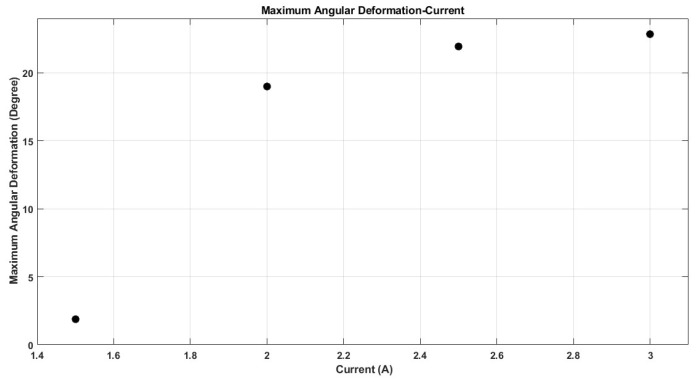
Maximum angular deformation of the front dome at each current value.

**Figure 27:**
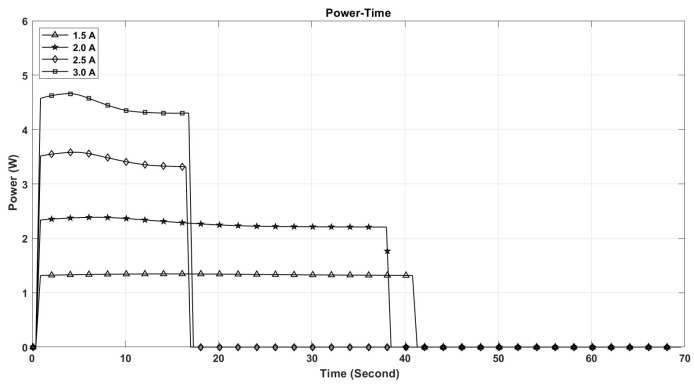
The power of the SMA spring actuator with time for different current values.

Figure 21, Figure 22, Figure 23, Figure 24, Figure 25, Figure 26 and Figure 27 present the experimentally measured performance of the proposed capsule prototype under different actuation conditions. These figures summarize the key experimental results obtained with the fabricated system, including dome manipulation, locomotion behavior, stroke response, angular deformation, and the thermal response of the SMA actuators. In particular, Figure 21 was included as a preliminary thermal characterization of the bare SMA actuator under different DC current inputs to identify an appropriate operating range for the proposed mechanism. It should be noted that the reported temperatures correspond to the SMA spring itself in the benchtop prototype and not to the external surface temperature of a fully packaged capsule. Although a current of 3 A provided the largest stroke and fastest response, the measured actuator temperature reached approximately 110 °C, which is not suitable for direct biomedical use without thermal isolation. From a safety perspective, lower current levels provide a safer and more conservative operating range and can be considered safer candidates for future implementation, particularly when combined with thermal insulation, encapsulation, duty-cycle control, and closed-loop temperature regulation.

The maximum stroke and angular deformation of the capsule increase with increasing current, achieving values of approximately 5.4 mm and 24 degrees at a current of 3 A, respectively, as illustrated in Figure 25 and Figure 26. While it is evident that the capsule’s response time enhances with an increase in current, it is important to note that the power consumption of the springs also escalates, as demonstrated in Figure 27.

The experimental prototype used in this study was fabricated at approximately 3.5× the scale of the intended clinical capsule dimensions to facilitate rapid prototyping and measurement, see the video in the Supplementary Materials. Consequently, the measured locomotion stroke of 5.4 mm corresponds to the scaled prototype. When geometrically scaled to the intended capsule size of 25×15 mm, the expected locomotion stroke is proportionally reduced to approximately 1.98 mm, as indicated by the FEA. It should be noted that the primary goal of the scaled prototype was to validate the working principle of the compliant mechanism and integrated locomotion–steering concept. While geometric scaling affects the absolute displacement, the kinematic behavior and actuator coordination remain representative of the proposed capsule architecture. Further miniaturization and optimization will be required to maximize displacement at the clinical scale. The improved locomotion performance can be attributed to the optimized compliant mechanism architecture and the efficient utilization of SMA spring actuators. In particular, the flexure hinge optimization enabled larger deformation while maintaining structural integrity, thereby increasing the achievable stroke without significantly increasing the system complexity. It is important to note that compliance behavior and thermal dissipation do not scale linearly. Therefore, the macro-scale prototype serves only as a proof-of-concept validation. Miniaturization to clinical dimensions will require re-optimization of hinge stiffness, SMA actuator sizing, and thermal isolation strategies.

SMA fatigue behavior is an important consideration for cyclic operation in capsule robots. In this work, the SMA springs were operated within recommended strain limits to ensure repeatable actuation. The primary objective of this study is to validate the proposed mechanism and integrated actuation concept. Detailed fatigue characterization and long-cycle durability testing of the SMA actuators will be investigated in future work.

As summarized in Table 1, the proposed capsule provides competitive locomotion performance while maintaining a compact architecture and integrating steering and propulsion using the same actuator set.

Table 5 summarizes the key experimental performance metrics of the scaled capsule system obtained from the locomotion and steering experiments.

The results summarized in Table 5 demonstrate that the proposed capsule architecture achieves effective locomotion and steering performance while maintaining a relatively simple actuation mechanism compared with many internally actuated capsule robots reported in the literature.

### Thermal Safety and Considerations for In Vivo Deployment

The thermal characteristics of the SMA actuators are another crucial consideration for the potential biomedical application of the proposed capsule endoscope. As depicted in Figure 21, the temperature of the SMA spring increases with the input current and reaches approximately 110 °C at an input current of 3 A. Although the highest stroke and speed are obtained at the highest input current, such high temperatures are not suitable for the direct application of the proposed capsule endoscope in biomedical applications.

From the clinical safety point of view, the general consensus for the safe temperature for the continuous contact of the proposed capsule endoscope for human tissue is that the tissue temperature needs to be kept below approximately 43 °C and, for short-term exposure, the tissue temperature needs to be kept below approximately 50 °C [45].

Although the SMA temperature reached up to 110 °C during open-air testing, the final capsule design incorporates multiple thermal isolation strategies to protect surrounding tissue. These include a low thermal conductivity polymer outer shell, internal air gaps between the SMA and capsule wall, localized insulation around the SMA springs, and pulsed actuation to limit heat accumulation. In addition, custom low-transition-temperature SMA elements will be used in the final design to reduce the required operating temperature.

The reported 5.4 mm stroke represents the mechanical output of the scaled prototype under controlled laboratory conditions. In an actual gastrointestinal environment, the effective locomotion performance will be influenced by additional factors such as variable friction, lumen diameter changes, wall compliance, curvature, and the presence of fluids or mucus. These factors may reduce the net forward displacement achieved per actuation cycle. Therefore, the present results should be interpreted as proof-of-concept validation of the proposed locomotion mechanism rather than final in vivo performance. Future work will include testing in gastrointestinal phantom environments with compliant walls, variable diameters, and lubricated surfaces to evaluate the system under more realistic physiological conditions.

## 6. Conclusions

Wireless capsule endoscopy is a non-invasive diagnostic technique used for imaging the gastrointestinal tract, primarily utilized for imaging anatomical regions that are particularly challenging to assess through traditional endoscopy, notably the small bowel. As passive capsule endoscopy has several limitations, including localization issues and the potential for obstruction within the GI tract, active capsule endoscopy offers a promising solution through its integrated locomotion system.

In this study, we developed and fabricated a novel compliant active capsule actuated by a quartet of shape memory alloy (SMA) spring actuators. This semi-soft capsule comprises rigid PLA links interconnected with TPU 92A flexible hinges (FHs). We conducted a nonlinear finite element analysis (FEA) for static structural assessment using ANSYS Workbench to establish an initial design framework for the capsule, followed by an optimization process. The optimization involved shape refinement to select the most efficient notch configuration for the flexible hinges and dimensional adjustments to determine the optimal thickness of these hinges, aiming to maximize stroke while remaining within the yield stress limits of the materials used (15.6 MPa) and maintaining a capsule diameter of 15 mm.

Through the nonlinear FEA, we identified the optimal notch shape for the FHs, concluding that circular FHs were best suited for the proposed gripper design. This choice was motivated by the simplicity and ease of fabrication of circular FHs, as well as their favorable stress distribution—attributed to the filleted regions at the ends—which enhances the fatigue life of the component. Additionally, the axial rotation shift for circular FHs was found to be minimal in comparison to other designs, ensuring greater precision in modeling.

Subsequently, we fabricated the optimized capsule design using Fused Deposition Modeling (FDM) 3D printing technology to validate the findings from the FEA and modeling analyses. The capsule was effectively connected to the SMA actuators and an Arduino UNO-controlled circuit, allowing for the assessment of its capabilities in forward movement, rotation (both right and left), and manipulation of the front dome. The experimental results support these functionalities, demonstrating their effectiveness and reliability throughout the study.

Future work will focus on miniaturization of the capsule architecture and experimental validation in gastrointestinal phantom environments to further evaluate its locomotion and steering performance under realistic physiological conditions.

## Figures and Tables

**Table 1:** Comparison of representative capsule endoscopy systems categorized by internal and external actuation.

Reference	Year	Actuation Method	Steering	Capsule Size (mm)	Locomotion	Actuators	Transmission	External Hardware	Fabrication	Complexity
**Passive Capsule**										
Wireless capsule endoscope [4]	2000	Passive (GI peristalsis)	No	26×11	None	0	None	None	Standard capsule assembly	Very Low
**Internal Actuation**										
Ciuti et al. [21]	2012	Vibratory motor	No	34×15 mm	Continuous motion	1 motor	Direct vibration	None	Standard capsule integration	Low
Chen et al. [42]	2014	Internal DC motor	Limited	45 × 16 mm	Crawling locomotion	1 DC motor	Spiral legs	None	Multi-part assembly	Medium
Simi et al. [30]	2010	Hybrid internal + magnetic	Yes	Diameter (14 mm)	Crawling locomotion	Internal actuator	Leg linkage	External magnet	Multi-part precision assembly	High
**External Magnetic Actuation**										
Lee et al. (ALICE) [43]	2014	Electromagnetic actuation	Yes	20×8 mm	Active locomotion	0 (internal propulsion)	Magnetic driven	External EM system	Multi-module assembly	High
Hoang et al. [44]	2022	External electromagnetic actuation	Yes	34.6×12.5 mm	Active locomotion	0 (internal propulsion)	Magnetic driven	EM coil system	Capsule + external setup	High
Yu et al. (Hybrid magnetic locomotion) [38]	2025	External permanent magnet	Yes	31.3×18 mm	Inchworm + spiral locomotion	Internal magnets	Magnetic torsion spring	External permanent magnet	Multi-module magnetic structure	High
Peker et al. [13]	2024	External electromagnets	Yes	25×13 mm	Continuous navigation	0 (internal propulsion)	Magnetic driven	Electromagnet platform	Capsule + external system	High
**Proposed SMA Capsule**										
**Prototype Capsule**	2026	**4 SMA spring actuators**	**Yes (integrated)**	**87.5×52.5 mm**	**∼5.4 mm stroke**	**4 SMA (shared)**	**Compliant mechanism**	**None**	**Dual-material 3D printing**	**Low**
**Proposed Capsule**	2026	**4 SMA spring actuators**	**Yes (integrated)**	**25×15 mm**	**∼1.5 mm stroke**	**4 SMA (shared)**	**Compliant mechanism**	**None**	**Dual-material 3D printing**	**Low**

**Table 2:** Mechanical properties of 3D printing materials.

Material	E (MPa)	ν	ρ (kg/m^3^)	Yield Strength (MPa)
**PLA**	3450	0.39	1250	54.1
**TPU 92A**	15.3	0.45	1135	15.6

**Table 3:** Thickness optimization parameters, constraints, objectives, and optimal values.

Optimization Parameters	Optimization Constraints		Optimization Objectives	Optimal Value
	Lower Bound	Upper Bound		
Upper & Lower FHs Minimum Thickness (ULTHh)	0.2 mm	1.2 mm	-	0.88 mm
Middle FHs Minimum Thickness (MTh)	0.2 mm	1.2 mm	-	0.52 mm
Upper FHs Von Mises Stress (UVS)	0 MPa	10 MPa	Minimize	2.3 MPa
Middle FHs Von Mises Stress (MVS)	0 MPa	10 MPa	Minimize	3.62 MPa
Lower FHs Von Mises Stress (LVS)	0 MPa	10 MPa	Minimize	2.3 MPa
Edge Deformation (ED)	0 mm	2 mm	-	1.97 mm
Capsule Stroke (S)	-	-	Maximize	-

**Table 4:** Primary settings for 3D printing of capsule parts.

Print Settings	PLA	TPU 92A
Layer thickness	0.2 mm	0.2 mm
Build plate temperature	60 °C	60 °C
Printing temperature	210 °C	240 °C
Printing speed	60 mm/s	30 mm/s
Fan speed	100%	100%
Infill density	20%	100%
Build plate adhesive	Skirt	Skirt

**Table 5:** Summary of the experimental performance of the scaled capsule prototype.

Parameter	Measured Value	Notes
Maximum locomotion stroke	5.4 mm	Obtained at 3 A input current
Maximum steering angle	24°	Angular deformation of front dome
Average locomotion speed	0.1–0.85 mm/s	Estimated from Figure 23
Operating current range	1.5–3 A	Experimental actuation range
SMA actuator temperature	45–110 °C	Measured using FLIR thermal camera (Tallinn, Estonia)
Prototype scale	3.5×	Scaled experimental prototype

## Data Availability

The data supporting the findings of this study are available from the corresponding author upon request.

## Supplementary Materials

The following supporting information can be downloaded at: https://www.mdpi.com/article/10.3390/mi17040471/s1, Video S1: Capsue Motion in Irregular Path.

## Abbreviations

The following abbreviations are used in this manuscript:

GI	Gastrointestinal
SMA	Shape Memory Alloy
PLA	Polylactic Acid
TPU	Thermoplastic Polyurethane
FH	Flexure Hinge
FEA	Finite Element Analysis
FDM	Fused Deposition Modeling
WCE	Wireless capsule endoscopy
FPS	Frames per second
FOV	Field of view
CMOS	Complementary metal-oxide semiconductor
LED	Light-emitting diode
CCD	Charged-coupled device
AFI	Auto-fluorescence imaging
NBI	Narrow-band imaging
MICS	Medical Implant Communications Service
ISM	Industrial, scientific and medical
MRI	Magnetic resonance imaging
RF	Radiofrequency
WPT	Wireless power transmission
MGCE	Magnetically Guided Capsule Endoscopy
EMA	Electromagnetic actuation
EM	Electromagnetic
MFN	Magnetic Field Navigator
DC	Direct Current
IPMC	Ionic polymer–metal composite
PDMS	polydimethylsiloxane
PFPE	perfluoropolyether
MEMS	Microelectromechanical
EDM	Electrical Discharge Machining
FFF	Fused Filament Fabrication
NiTiCu	Nickel–Titanium–Copper
DAQ	Data Acquisition
POI	Point of Interest
